# Targeting Thyrointegrin αvβ3 Using Fluorobenzyl Polyethylene Glycol Conjugated Tetraiodothyroacetic Acid (NP751) in Acute Myeloid Leukemia

**DOI:** 10.3389/fonc.2021.793810

**Published:** 2022-01-27

**Authors:** Noureldien H. E. Darwish, Gennadi V. Glinsky, Thangirala Sudha, Shaker A. Mousa

**Affiliations:** ^1^ Pharmaceutical Research Institute, Albany College of Pharmacy and Health Sciences, Rensselaer, NY, United States; ^2^ Hematology Unit, Clinical Pathology Department, Faculty of Medicine, Mansoura University, Mansoura, Egypt; ^3^ Institute of Engineering in Medicine, University of California San Diego, San Diego, CA, United States

**Keywords:** thyrointegrin αvβ3, molecular mechanism, AML management, acute myeloid leukemia, AML

## Abstract

**Background:**

Acute myeloid leukemia (AML) is associated with poor long-term survival, even with newer therapeutic agents. Here, we show the results of our preclinical study, in which we evaluated the efficacy of a new thyrointegrin αvβ3 antagonist, named fluorobenzyl polyethylene glycol conjugated tetraiodothyroacetic acid (fb-PMT).

**Methods and Results:**

fb-PMT (NP751) is a potent αvβ3 antagonist of molecular weight of 2,478.9 Da. it represents a conjugate of tetraiodothyroacetic acid (tetrac) and monodisperse polyethylene glycol (PEG36), with a 4-fluorobenzyl group capping the other end of the PEG. fb-PMT effectively suppresses the malignant growth of human acute myeloid leukemia (AML) after successful engraftment in transgenic NSG-S xenograft mouse models of either established human AML cell line or primary AML cells. Daily treatment with fb-PMT (1–10 mg/kg body weight) subcutaneously (s.c.) for 3–4 weeks was associated with marked regression of leukemogenesis and extended survival in both models. The efficiency of the fb-PMT therapy was verified using *in vivo* imaging system (IVIS) imaging, flow cytometry, and histopathological examination to monitor the engraftment of leukemic cells in the bone marrow and other organs. fb-PMT therapy for 3–4 weeks at 3 and 10 mg/kg daily doses exhibited significant reduction (*p* < 0.0001) of leukemic cell burden of 74% and >95%, respectively. All fb-PMT-treated mice in the 10 mg/kg treatment arm successfully maintained remission after discontinuing the daily treatment. Comprehensive fb-PMT safety assessments demonstrated excellent safety and tolerability at multiple folds above the anticipated human therapeutic doses. Lastly, our genome-wide microarray screens demonstrated that fb-PMT works through the molecular interference mechanism with multiple signaling pathways contributing to growth and survival of leukemic cells.

**Conclusion:**

Our preclinical findings of the potent anticancer activities of fb-PMT and its favorable safety profiles warrant its clinical investigation for the effective and safe management of AML.

## Introduction

Acute myeloid leukemia (AML) is one of the most aggressive malignant hematological disorders. More than 20,000 new cases were estimated to occur in the United States in 2021 (33.0% of all kinds of new leukemia cases) (1). In 2021, the estimated number of deaths from AML is 11,400, which represents 56.32% of new cases (1.0% of all cancer deaths), with an overall 5-year survival rate of 27%. Average age of diagnosis with AML is about 68 years (1). Current treatment regimens for AML include traditional chemotherapy, allogeneic hematopoietic cell transplantation, and targeted therapies for specific mutations in limited numbers of AML patients [e.g., Midostaurin, a FLT3 inhibitor, first gene mutation-targeted therapeutic agent approved by Food and Drug Administration (FDA) 2017], all of whom still suffer from adverse effects and relapse (2, 3). New broad spectrum effective and safe treatment options are urgently needed for the different types of AML.

According to the World Health Organization report in 2019, AML is now curable in up to 40% of patients under the age of 60 years and in up to 15% of patients over the age of 60. In elderly patients who are unable to tolerate the available chemotherapy, mean survival is 5–10 months (1, 4). Important advances in defining the “genomic landscape” of AML have enabled the prognostic classification of the disease and, to some extent, have facilitated the choice of chemotherapeutic agents. Among the mutations of particular interest and importance in AML are those of the *FLT3*, *NPM*, *CEBPA*, *KIT*, *NRAS*, *IDH1/2*, and *TP53* genes (5). Targeted therapies for specific mutations in treating AML were recently approved by the FDA based on the genetic basis of the disease in different subsets of AML patients. However, those targeted therapies treat only a small percentage of the AML population, and patients suffer from relapse upon discontinuation of treatment (3).

On the other hand, crosstalk between leukemic cells and the bone marrow microenvironment is recognized as one of important factors that keeps leukemic cells in quiescent state and helps them to escape the different therapies. One of the functionally important entities in the bone marrow microenvironment is represented by the integrins. Integrins are a family of heterodimeric structural proteins of the plasma membrane that are important to cell–cell interactions and cell motility (6, 7). They are critical to leukocytes’ function and to the angiogenesis process, particularly local release of growth factors that are generously overexpressed and produced by cancer cells and by dividing endothelial cells of tumor-relevant blood vessels. There are different members of the integrin family including αvβ3, αvβ5, and α5β1 that are involved in angiogenesis and have high affinity to arginine–glycine–aspartate (RGD) extracellular matrix (ECM)-containing angiogenesis modulators such as fibronectin, fibrinogen, and ostepontin (8–10). αvβ3 (thyrointegrin) is generously expressed by cancer cells and by dividing endothelial cells. Mechanistically, thyrointegrin αvβ3 enhances β-catenin signaling and the tyrosine kinase activities in AML (11, 12). Integrin αvβ3 was reported to be highly expressed in AML and associated with poor outcomes (13, 14).

This study focused on comprehensive preclinical evaluations of anticancer activities of a novel thyrointegrin αvβ3 antagonist, fb-PMT in models of AML. Mechanistically, fb-PMT and similar molecules bind to a specific site on thyrointegrin αvβ3 receptors that can be blocked with high affinity by various triazole tetrac derivatives (15–18). Our genome-wide microarray screens demonstrated that fb-PMT appears to exert its potent anticancer actions on human AML through a molecular interference mechanism with multiple signaling pathways supporting growth and survival of leukemic cells.

## Materials and Methods

### Tumor Cells and Test Compound

AML cell lines (K562-Luc; human erythroleukemia cells and KG1a; human myelocytic leukemia cells) were obtained from American Type Culture Collection (ATCC, Manassas, VA, USA). These cell lines are commonly used in research studies as experimental AML models (19–21). Primary human AML cells (*de novo* AML 6373, harboring FLT3-ITD mutation) were collected by leukapheresis from AML patients at the University Hospital, University of Pennsylvania, with informed consents (IRB protocol 703185).

K562-Luc cells were maintained in Roswell Park Memorial Institute (RPMI) 1640 media supplemented with 10% fetal bovine serum (FBS). To maintain luciferase-labeled cells, blasticidin antibiotic was added at a concentration of 8 µg/ml.

In our experiments, KG1a cells were cultured in RPMI 1640 media supplemented with 10% fetal bovine serum. Both K562-Luc and KG1a cells were grown in a humidified chamber with 5% CO_2_ and 95% humidity at 37°C.

Fluorobenzyl polyethylene glycol mono-triazole tetraiodothyroacetic acid (fb-PMT) was synthesized at our facility (Pharmaceutical Research Institute, Rensselaer, NY, USA), and GMP scaleup was done at Dalton Pharmaceuticals (Toronto, Canada) (9, 18). fb-PMT (molecular weight 2,478.9 Da) is a white powder that is soluble in Tris buffer at pH 8.0, ~100 mg/ml, with a final pH of 7.4.

### Competitive Binding of fb-PMT to Purified αvβ3

The binding affinity of fb-PMT to purified αvβ3 was assessed using a previously described methods (9, 22). Purified αvβ3 (1 μg/ml) was coated to polystyrene microtiter plate wells at 4°C overnight, and then, the wells were blocked with 3% bovine serum albumin (BSA) for 2 h at room temperature. The wells were washed with buffer A (50 mM Tris/HCl, 100 mM NaCl, 1 mM CaCl_2_, 1 mM MgCl_2_, 1% BSA), and anti-αvβ3 conjugated with biotin (1:1000 in buffer A) was added and incubated for 1 h at room temperature. Increasing concentrations of compounds were added in the presence or absence of fibrinogen and incubated for 2 h at room temperature, and then, wells were washed three times with buffer A and incubated with a streptavidin−horseradish peroxidase (HRP) conjugate (1:1,000 in buffer A) for 1 h at room temperature. Finally, wells were washed three times with buffer A, and 100 μl peroxidase substrate 3,3′,5,5′-tetramethylbenzidine (TMB) was added, and the reaction was terminated after 30 min with 50 μl of 450 nm stop solution for TMB. Absorbance was determined at 450 nm with a plate reader.

### Animals and Treatment Protocols

Animal studies were carried out at the Albany Stratton VA Medical Center (Albany, NY, USA) animal facility, and protocols were approved by the Institutional Animal Care and Use Committee (IACUC) (protocol number 545017). Eighty male NSG-S mice (6–8 weeks of age) were purchased from Jackson Laboratories (Bar Harbor, ME). Preconditioning was done by intraperitoneal injection of busulfan (30 mg/kg, Otsuka America Pharmaceutical Inc., Hayward, CA, USA) 24 h prior to cell injections. K562-Luc cells and primary AML cells (6373) (5–10 × 10^6^ per mouse) were transplanted *via* tail vein injection into mice.

For the K562-Luc animal model (40 mice), *in vivo* imaging system (IVIS) (Perkin Elmer, Waltham, MA, USA) scans and the peripheral blood smears examination were performed on animals once a week. The fb-PMT treatment schedule was initiated on day 10 post-implantation when increased counts of blast cells became evident in peripheral blood smears and confirmed by IVIS signals. fb-PMT was administered subcutaneously (s.c.) daily at three different doses (1, 3, and 10 mg/kg body weight) or vehicle, phosphate-buffered saline (PBS) (control) for 21 days for both the ON arm (21 days treatment and then 20 mice have been sacrificed) and the ON+OFF arm (21 days treatment followed by 14 days treatment discontinuation, and then, the remaining 20 mice have been sacrificed). Control animals were administered with vehicle (PBS, pH 7.4) daily.

In experiments using the primary AML cell animal model (40 mice), the treatment protocol was initiated on animals after confirmation of successful engraftment. Treatment was initiated on day 40 post-implantation with fb-PMT (1, 3, and 10 mg/kg) or control (vehicle, PBS) daily s.c. for 28 days. Twenty animals (ON arm) were humanely sacrificed after 28 days of treatment, and peripheral blood smears and bone marrow aspirates were examined histologically at the end of the 28 days of treatment. To evaluate the relapse after treatment, the remaining 20 mice (ON + OFF arm) were maintained without treatment for an additional 14 days; then, they were humanely sacrificed and processed to obtain samples of peripheral blood smears, bone marrow aspirates, and organs for histological examination. Maintaining for 14 days (ON + OFF) was established based on the animals’ condition in the control groups.

### Assessment of Leukemic Cells Engraftment by Flow Cytometry and Immunohistostaining

Human AML engraftment was assessed by flow cytometry and defined as the percentage of human CD45+/CD33+ cells in total live mononuclear cells. Fresh bone marrow cells from NSG-S mice engrafted with K562-Luc and primary AML cells (6373) were collected at day 10 and 40 post-engraftment, respectively, once the blast cells were detected in peripheral blood smear. Samples were stained with antibodies for cell surface markers: anti-human CD45-PE and anti-human CD33-FITC (BD Biosciences, San Jose, CA, USA). Cells were incubated with monoclonal antibodies for 15 min at room temperature, washed once in PBS containing 0.1% human serum albumin (HSA), and analyzed by flow cytometry. Data acquisition was performed using a FACS Aria III (BD Biosciences) equipped with an argon and red diode laser, and analysis was performed using Cell Quest software (BD Biosciences).

As the K562-Luc cells express dim CD34, immunohistochemistry was performed for the formalin-fixed decalcified femurs from primary AML cells (6373) transplanted mice, paraffin-embedded and sectioned at 5-µm sections. Slides were stained using human anti-CD34 primary antibody (R&D System, Minneapolis, MN, USA), then with HRP-conjugated secondary antibody (Cell Signaling Technology Inc. Danvers, MA, USA). HRP activity was detected by diaminobenzidine tetrahydrochloride (DAB), and the slides were counterstained by methyl green.

### RNA Isolation From AML Cells and Microarrays

K562-Luc and KG1a cells were cultured in 50-cm^2^ cell culture flasks with 10 ml phenol red free RPMI media containing 10% fetal calf serum (FCS) to 75% confluence. K562-Luc and KG1a cells were treated (at 50% confluence) with 30 µM fb-PMT for 48 h. Total RNA was immediately isolated from harvested cells using Trizole and checked for quality using an Agilent Bioanalyzer (Agilent Technologies, Santa Clara, CA) before being used for microarray analysis. The quality and the concentrations of the extracted RNA were analyzed using a NanoDrop (Thermo Fisher Scientific, Waltham, MA) and the Agilent Bioanalyzer. RNA samples (100 ng) deemed to be of sufficient quality (RIN >8) were processed according to the standard Affymetrix RNA labeling protocol. At least two independent biological replicates of control and treated samples were concurrently interrogated in microarray analyses. In preliminary experiments, the treatment dose and duration were carefully selected so as to not significantly affect growth and survival of target cells for the duration of the experiments.

### Microarray Analysis

Labeled RNA samples were processed for hybridization employing the Clariom™ S human array platform (Affymetrix, Santa Clara, CA) at the Center for Functional Genomics, University at Albany, Rensselaer, NY. Briefly, 100 ng of total RNA was processed using the WT Plus Reagent kit (Affymetrix). Sense target complementary DNAs (cDNAs) were generated using the standard Affymetrix WT protocol and hybridized to Affymetrix Human Clariom S arrays. Arrays were washed, stained, and scanned on a GeneChip 3000 7G scanner using Affymetrix GeneChip Command Console Software (AGCC). Transcriptome Analysis Console Software (TAC v3.0.1.5) was used to identify differentially expressed genes (DEGs). Briefly, the CEL files were summarized using the SST-RMA algorithm in TAC, and the normalized data were subjected to one-way ANOVA with a Benjamini–Hochberg false discovery rate correction included (*p* < 0.05). A 1.5-fold expression change cutoff was used to select entities that were statistically differentially expressed between the conditions being compared (treated and untreated groups). In the standard workflow protocol, the fragmented biotin-labeled cDNAs were hybridized for 16 h to Affymetrix Arrays, scanned on an Affymetrix Scanner 3000 7G using AGCC software, and processed as described above. Alternatively, CEL files after QC screening using Affymetrix Expression Console software were imported into GeneSpring GX11.5 (Agilent Technologies). The data were then quantile normalized using PLIER and baseline transformed to the median of the control samples. The probe sets were further filtered to exclude the bottom 20th percentile across all samples. The resulting entity lists were subjected to an unpaired t-test with the Benjamini–Hochberg false discovery rate correction and a 1.5-fold expression changes filter to identify differentially expressed transcripts between the control and test conditions at a *p <*0.05. All analyzed and reported data are MIAME compliant, and the raw data have been deposited in Gene Expression Omnibus (GEO; GSE95790) as detailed on the Microarray Gene Expression Data Society (MGED) website (http://www.mged.org/Workgroups/MIAME/miame.html). Overall, the workflow of the microarray analyses was modeled based on previously published contributions (23).

Gene set enrichment analyses of DEGs were done using the Enrichr bioinformatics platform, which enables the interrogation of nearly 200,000 gene sets from more than 100 gene set libraries. The Enrichr API (January 2018 through October 2020 releases) (24, 25) was used to test genes of interest for significant enrichment in numerous functional categories. When technically and analytically feasible, different sets of DEGs defined at multiple significance levels of statistical metrics and comprising dozens to several thousand individual genetic loci were analyzed using differential gene set enrichment analysis (GSEA) to gain insights into biological effects of DEGs and infer potential mechanisms of anticancer activities. This approach was successfully implemented for the identification and characterization of human-specific regulatory networks governed by human-specific transcription factor-binding sites (26) and functional enhancer element (27): 13,824 genes associated with 59,732 human-specific regulatory sequences (28) and 8,405 genes associated with 35,074 human-specific neuroregulatory single-nucleotide changes (29). Initial GSEAs entail interrogations of each specific set of DEGs using 29 distinct genomic databases, including comprehensive pathway enrichment Gene Ontology (GO) analyses followed by in-depth analyses of the selected genomic databases deemed most statistically informative. In all tables and plots (unless stated otherwise), in addition to the nominal *p*-values and adjusted *p*-values, the “combined score” calculated by Enrichr software is reported, which is a product of the significance estimate and the magnitude of enrichment [combined score c = log(p) * z, where p is the Fisher’s exact test p-value and z is the z-score deviation from the expected rank].

### Statistical Analysis

An overall comparison of the means for all groups was carried out using a one-way ANOVA. Tukey confidence intervals were used to test for differences in means for each experimental group versus the control group. Results are presented as means ± SD. A value of *p* < 0.05 indicated a statistically significant difference.

## Results

### *In Vitro* Binding Affinity of fb-PMT With the Thyrointegrin αvβ3

**Figure 1:**
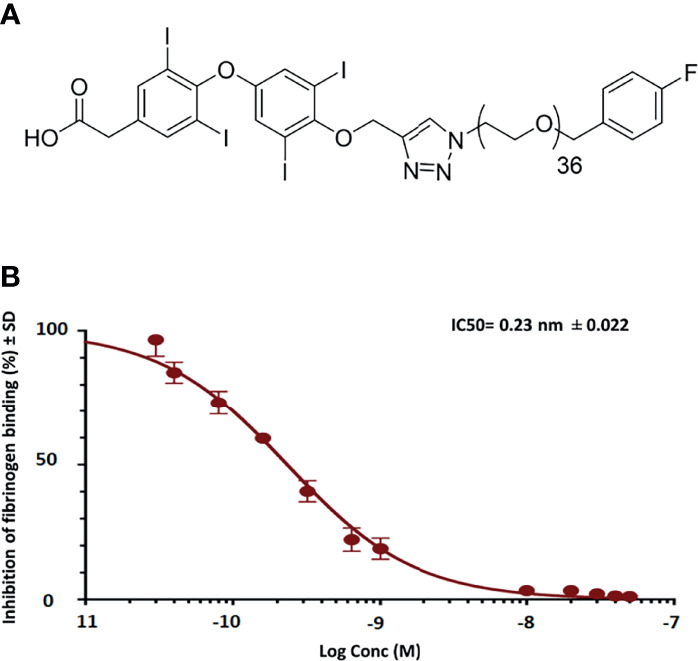
Representative figures show schematic structure of fb-PMT (molecular weight 2,478.9 Da) **(A)** and binding affinity of fb-PMT to human integrin avb3 receptors. fb-PMT manifests high affinity for the thyrointegrin αvβ3 receptors (Ki 0.23 nM) **(B)**.

In binding affinity experiments of fb-PMT, we confirmed that fb-PMT has a high affinity for the thyrointegrin αvβ3 receptors with a lower IC_50_ (50% inhibitory concentration) of 0.23 nM (**Figure 1**).

### Preclinical *In Vivo* Therapy Experiments Revealed Potent fb-PMT Anticancer Activities

#### Effects of fb-PMT Therapy on K562-Luc Human Leukemic Cell Line and FLT3-ITD Primary Human AML Cells Engrafted in Transgenic Mice

**Figure 2:**
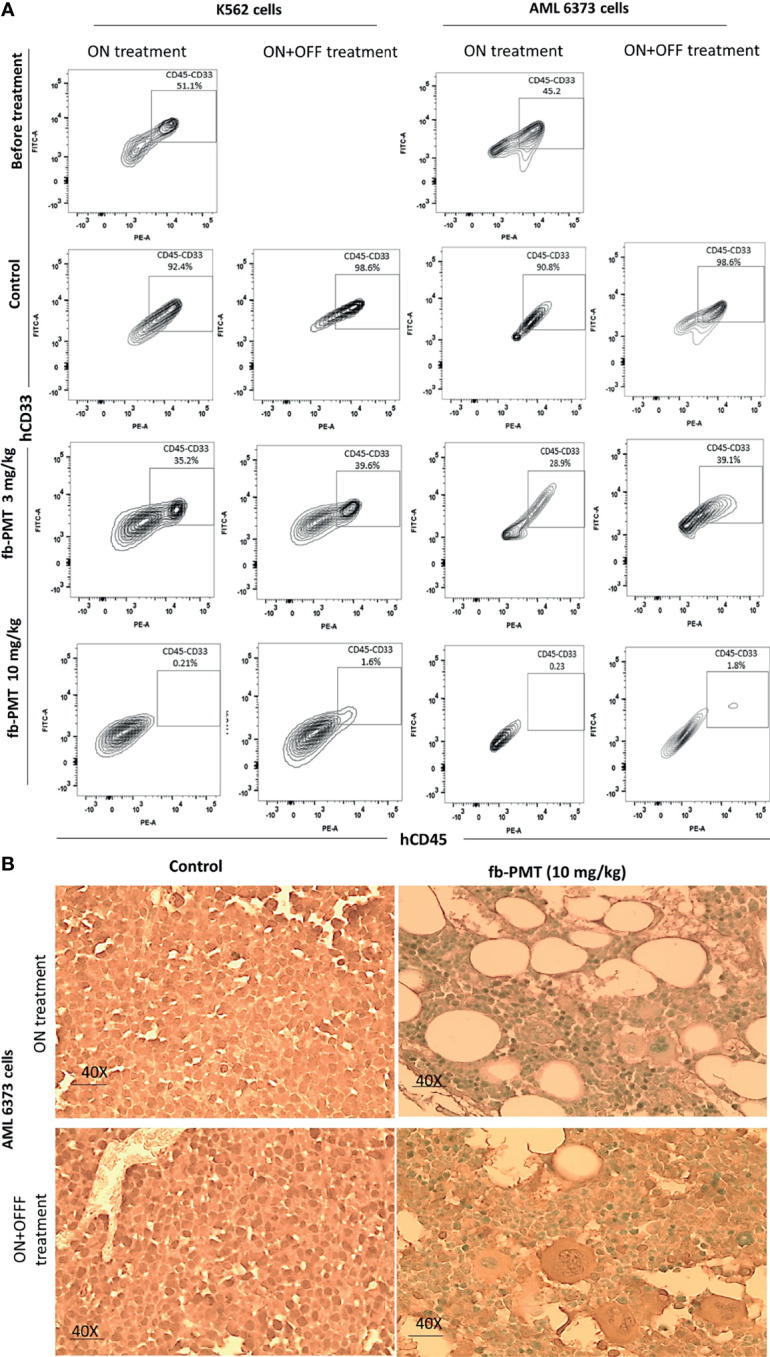
Leukemic cells (k562-Luc and primary AML cells, 6373) engraftment. Leukemic cells engraftment is markedly affected by fb-PMT (10 mg/kg). **(A)** Flow cytometry plots of human hCD45+/CD33+ leukemia cells from the NSG-S mice bone marrow samples: before treatment, after treatment in ON and ON + OFF treatment group [Control, fb-PMT (3 and 10 mg/kg)]. Bone marrow engraftment with k562-Luc and primary AML cells, 6373 (51.1% and 45.2%) was confirmed before starting treatment 10 and 40 days post-implantation, respectively. Elevated levels of human hCD45+/CD33+ leukemia cells were seen in NSG-S mice in control groups in comparison to treated groups. **(B)** hCD34 immunohistostaining of paraffin-embedded bone marrow sections from of mice engrafted with 6373 primary AML cells. Control group in both ON and ON + OFF group shows hypercellular BM with hCD34+ leukemic cells (>95%). fb-PMT (10 mg/kg) treatment groups show regeneration of bone marrow with normocellular bone marrow and absent of hCD34+ cells.

In the K562-Luc engrafted in transgenic mice, blast cells appeared in the blood smears of NSG-S mice 10 days after engraftment, with an average value of 40%. After 21 days of fb-PMT daily s.c. injection, blast cell counts continually and consistently decreased in a dose-dependent manner in treated versus control groups, while animals in the control group showed increased blast cells in the peripheral blood. No blast cells could be detected in fb-PMT-treated animals (10 mg/kg) at the end of the treatment. Furthermore, there was no rebound increase in peripheral blast cells at 1 and 3 mg/kg with full sustained remission at fb-PMT dose of 10 mg/kg at 1–2 weeks post-discontinuation of treatment (**Figure 2**; **Supplementary Figure S1A**).

On the other hand, primary AML cells (6373-FLT3-ITD) cells appeared in the blood smears of NSG-S mice 40 days after engraftment, with an average value of 26%. After 28 days of daily s.c. treatment, peripheral smears of treated animals were entirely normal at fb-PMT dose of 10 mg/kg. Daily s.c. injections of fb-PMT at 1, 3, and 10 mg/kg doses prevented blast cell expression/reproduction compared to controls by 54%, 75%, and 98.5%, respectively (**Figure 2**; **Supplementary Figure S1B**).

**Figure 3:**
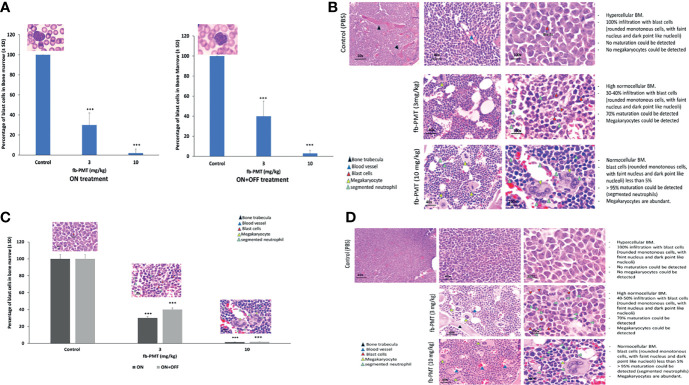
Blast cells in bone marrow. **(A)** Myeloblast in bone marrow of transgenic mice engrafted with K562-Luc cells and after 21 days of treatment (ON treatment) and 14 days post-treatment (ON + OFF treatment). Bone marrow of fb-PMT (3 mg/kg) treated group was associated with 30%–40% infiltration with blast cells, and 70% maturation could be detected. fb-PMT (10 mg/kg) treated group presented with blast cells <5% and 90%–95% normal maturation (segmented neutrophils). ON + OFF treatment, fb-PMT (10 mg/kg) showed maintained remission. **(B)** Histopathological evaluation of bone marrow of transgenic mice engrafted with K562-Luc cells (ON + OFF treatment). Myeloblast in bone marrow of transgenic mice after 21 days of treatment. Bone marrow of fb-PMT (3 mg/kg) treated group was associated with 30%–40% infiltration with blast cells, and 70% maturation could be detected. fb-PMT (10 mg/kg) treated group presented with blast cells <5% and 90%–95% normal maturation (segmented neutrophils). **(C)** Myeloblast in bone marrow from transgenic mice engrafted with primary AML cells (6373-FlT3-ITD) control versus fb-PMT-treated animal through 28 and 14 days post-treatment. More than 20% immature blast cells in marrow smears is characteristic of AML. Treated group (10 mg/kg) was associated with >90% decrease in leukemic cells burden. The ON + OFF group showed sustained remission with fb-PMT (10 mg/kg) after 14 days. **(D)** Histopathological evaluation of bone marrow in transgenic mice engrafted with primary AML cells (6373-FlT3-ITD) control versus fb-PMT-treated animal through 28 and 14 days post-treatment. Bone marrow of fb-PMT (3 mg/kg) treated group was associated with 35% infiltration with blast cells, and 60%–70% maturation could be detected. The fb-PMT (10 mg/kg) treated group presented with blast cells <5% and >95% normal maturation (segmented neutrophils) (***p < 0.0001.

After termination, the bone marrow K562-Luc engrafted mice with a daily treatment of fb-PMT at 3 mg/kg manifested 30%–40% infiltration with blast cells, while 70% maturation could be detected. fb-PMT-treated animals at 10 mg/kg dose presented bone marrows with blast cell counts <5% and >95%; normal maturation have been documented (segmented neutrophils). The remission was maintained in all treated mice at least 2 weeks after fb-PMT therapy discontinuation (**Figures 3A, B**; **Supplementary Figure S2A**).

IVIS scans and histopathological results at sacrifice showed a dose-dependent decrease in the brain, lung, liver, and spleen infiltration with the leukemic cells in the group of fb-PMT-treated mice (10 mg/kg) in comparison to control group (**Supplementary Figures S3**, **S4**). The fb-PMT therapy at 10 mg/kg dose in the ON + OFF treatment group resulted in successfully maintained remission in all animals 2 weeks after withdrawal of the daily treatment. The sustained remission was confirmed using blood smear analyses, IVIS scans, flow cytometry, and histopathological examinations.

Regarding the bone marrow samples from mice engrafted with primary AML cells (FLT3-ITD), the fb-PMT-treated group (10 mg/kg) restored the normal bone marrow maturation with abundant megakaryocytes in comparison to control animals (**Figures 3C, D** **)**. The results were confirmed with flow cytometry and immunohistochemistry analysis (**Supplementary Figure S2**).

Furthermore, we evaluated the splenic infiltration in our animal models. Histopathological results showed a marked decrease in splenic metastases of the leukemic cells in the group treated with fb-PMT (10 mg/kg) compared to control group (**Supplementary Figure S5**). Similarly, to the K562-Luc AML experiments, the primary AML model (ON + OFF) group (10 mg/kg) manifested the successful maintenance of remission 2 weeks after withdrawal of daily therapy. The splenic weight showed marked decrease (80%) even with the low dose (1 mg/kg). The ON + OFF groups maintained normal splenic weight in comparison to control, which may reflect successful prevention of engraftment (**Supplementary Figure S5A**). Treatment with fb-PMT daily for 28 days (ON treatment) or 28 days ON and 14 days off treatment (ON + OFF treatment) resulted in maximal suppression of primary AML infiltration into the spleen of AML-engrafted transgenic mice (**Supplementary Figure S5B** **).**

Additionally, in our *in vivo* therapy experiments (fb-PMT), survival of the animals in the therapy group was 100% at the end of the treatment protocol. In the ON + OFF arm, all animals in the control group reached the moribund state (per definition of the experimental protocol approved by the IACUC) and must be humanly sacrificed. Thus, 100% of animals in the control group reached this terminal survival stage. Correspondingly, fatality rate in the control group was 100%, and survival rate in the therapy group was 100%. Finally, we did not notice any changes in mice weights in all treated groups in comparison to the control (untreated) (**Supplementary Figure S6** **).**

### Microarray Analyses of fb-PMT Effects on Gene Expression in Human AML Cells

We performed the microarray study after 24 h treatment with 30 μM fb-PMT [cell vitality 50%–70% after using co-culture of leukemic cells with bone marrow stroma cells (HS-5, ATCC)]. RNA samples were extracted from biological replicates of control and treated human AML cells and subjected to microarray analyses *Materials and Methods*. Results of these analyses are reported in **Tables 1**–**3** and **Supplementary Figures S7–S11** and **Supplementary Tables S1–S3**.

**Table 1:** Examples of mimicry of pathway activation and pathway interference gene expression signatures (GES) identified in fb-PMT-treated K562 cells.

fb−PMT pathway activation GES	Genes	*p*-Value*	Odds Ratio*	Combined Score*
RB1 pathway signature (n = 10)	STARD4;TMEM2;SESN3;SPIN4;SLX4IP;CD109;CENPE;CEP135;TCP11L2;PLD1	3.70E−04	6.266319	49.51741
IRF9 pathway signature (n = 6)	CENPE;EID3;SESN3;CD109;ASB7;TCP11L2	5.12E−04	7.54717	57.18613
MAML1 pathway signature (n = 6)	CENPE;PTAR1;STARD4;TMEM30A;CD109;TCP11L2	0.001655	4.696673	30.07697
RAP1A pathway signature (n = 5)	CENPE;SPIN4;ACSL1;ELOVL7;IL18R1	0.001067	6.410256	43.86473
GATA4 pathway signature (n = 5)	ERRFI1;ACSL1;LRRC28;CD9;ELOVL7	4.15E−04	7.905138	61.56706
**fb-PMT pathway interference GES**	**Genes**	*p*-Value*	Odds Ratio*	Combined Score*
MYC pathway signature (n = 8)	ERRFI1;CENPE;BLOC1S6;TMEM30A;CD109;SESN3;ZNF17;KDSR	2.04E−04	9.21659	78.29477
HIF1A pathway signature (n = 6)	ERRFI1;ERV3-1;TCP11L2;SH3BGRL2;ELOVL7;IL18R1	1.48E−04	7.430341	65.52441
TWIST1 pathway signature (n = 5)	EID3;SESN3;CD109;TCP11L2;ASB7	0.00152	5.91716	38.39501
TFAP2C pathway signature (n = 5)	CENPE;SPIN4;ACSL1;CD109;ELOVL7	5.21E−04	7.518797	56.84253

GES were identified based on the analyses of top 50 downregulated genes in fb-PMT-treated K562 cells.

*Statistical metrics were defined by the Enrichr bioinformatics platform (Materials and Methods).

**Table 2:** Mimicry of pathway activation and pathway interference gene expression signatures (GES) identified in fb-PMT-treated K562 cells.

Enriched Terms	*p*-Value*	Adjusted *p*-Value*	Odds Ratio*	Combined Score*	Genes
**IRF9 OE HUMAN GSE50002 CREEDSID GENE 1659 DOWN**	2.89E−07	5.65E−04	31.44654	473.5	*CENPE;EID3;SESN3;CD109;ASB7*
**TFAP2C SIRNA HUMAN GSE15481 CREEDSID GENE 2895 DOWN**	2.94E−07	2.88E−04	31.32832	471.2	*CENPE;ACSL1;SPIN4;CD109;ELOVL7*
**TFAP2C KD HUMAN GSE15481 CREEDSID GENE 2970 DOWN**	3.60E−07	2.35E−04	30.08424	446.4	*CENPE;ACSL1;SPIN4;CD109;ELOVL7*
**TWIST1 OE MOUSE GSE50002 CREEDSID GENE 1075 UP**	9.62E−07	4.71E−04	24.65483	341.6	*EID3;SESN3;CD109;ASB7;TCP11L2*
**IRF9 OE HUMAN GSE50002 CREEDSID GENE 1663 DOWN**	1.16E−06	4.54E−04	23.74169	324.5	*EID3;SESN3;CD109;ASB7;TCP11L2*
**MYC OE U2OS HUMAN GSE59819 RNASEQ UP**	6.23E−06	0.002034	30.72197	368.2	*CENPE;ERRFI1;TMEM30A;CD109*
**MYC OE U2OS HUMAN GSE66789 RNASEQ UP**	6.23E−06	0.001743	30.72197	368.2	*CENPE;ERRFI1;TMEM30A;CD109*
**IRF9 OE HUMAN GSE50002 CREEDSID GENE 1656 DOWN**	8.26E−06	0.002021	28.6123	334.9	*SESN3;CD109;ASB7;TCP11L2*
**IRF9 OE HUMAN GSE50002 CREEDSID GENE 1653 DOWN**	1.43E−05	0.00312	24.87562	277.4	*SESN3;CD109;ASB7;TCP11L2*
**IRF9 OE HUMAN GSE50002 CREEDSID GENE 1657 DOWN**	2.20E−05	0.004317	22.29654	239.1	*SESN3;CD109;ASB7;TCP11L2*
**IRF9 OE HUMAN GSE50002 CREEDSID GENE 1654 DOWN**	2.45E−05	0.004353	21.71553	230.6	*SESN3;CD109;ASB7;TCP11L2*
**HIF1A KO MOUSE GSE35111 CREEDSID GENE 1406 DOWN**	2.98E−05	0.004868	20.63983	215.1	*ERRFI1;TCP11L2;ELOVL7;IL18R1*

GES were identified based on the analyses of 12 downregulated genes in fb-PMT-treated K562 cells.

*Statistical metrics were defined by the Enrichr bioinformatics platform (Materials and Methods).

**Table 3:** Examples of functionally-significant genes downregulated by the fb-PMT treatment in KG1A human AML cells.

Description	Gene Symbol	Fold Change	*p*-Value
**X-linked inhibitor of apoptosis, E3 ubiquitin protein ligase**	*XIAP*	−2.9	0.0185
**Osteoclast stimulating factor 1**	*OSTF1*	−2.86	0.0331
**Signal transducer and activator of transcription 2**	*STAT2*	−2.81	0.0202
**thymopoietin**	*TMPO*	−2.59	0.0069
**Signal transducer and activator of transcription 4**	*STAT4*	−2.54	0.0451
**YES proto-oncogene 1, Src family tyrosine kinase**	*YES1*	−2.49	0.0315
**Polymerase (RNA) II (DNA directed) polypeptide B, 140kDa**	*POLR2B*	−2.44	0.0354
**Cyclin-dependent kinase 14**	*CDK14*	−2.21	0.0402
**TTK protein kinase**	*TTK*	−2.2	0.046
**Topoisomerase (DNA) III alpha**	*TOP3A*	−2.12	0.0116
**Prohibitin 2; small Cajal body-specific RNA 12**	*PHB2*	−2.03	0.042
**cyclin-dependent kinase 17**	*CDK17*	−1.97	0.0303
**phosphoglycerate kinase 2**	*PGK2*	−1.85	0.0471
**Polymerase (DNA directed), epsilon 2, accessory subunit**	*POLE2*	−1.83	0.0441
**B-Raf proto-oncogene, serine/threonine kinase**	*BRAF*	−1.81	0.0315
**Growth factor receptor bound protein 2**	*GRB2*	−1.81	0.0301
**Pim-1 proto-oncogene, serine/threonine kinase**	*PIM1*	−1.71	0.038
**Serine/threonine kinase 4**	*STK4*	−1.69	0.0135
**Prostaglandin E synthase 3 (cytosolic)**	*PTGES3*	−1.56	0.0429
**B-cell CLL/lymphoma 9**	*BCL9*	−1.52	0.0334

Overall, there were 518 significantly downregulated gene expression records and 283 significantly upregulated gene expression records, expressions of which were changed at least 1.5-fold in fb-PMT-treated K562 cells. In fb-PMT-treated KG1a cells, 223 significantly downregulated gene expression records and 191 significantly upregulated gene expression records were identified, expressions of which were changed at least 1.5-fold in fb-PMT-treated cells. All DEGs identified by the Affymetrix Expression Console software were subjected to GSEA employing a panel of 29 genomic databases *Materials and Methods*. Analyses of fb-PMT treatment-induced DEGs in both K562 and KG1a cells identified sets of downregulated genes and a genomic database of Transcription Factor (TF) Perturbations Followed by Expression as the most informative setting among comparison records. GSEA of downregulated DEGs using the TF Perturbations Followed by Expression database identified 60 and 84 significantly enriched records (adjusted *p* < 0.05) for fb-PMT-treated K562 and KG1a cells, respectively.

#### GSEA of fb-PMT Effects on Gene Expression Revealed Signatures of the Molecular Mimicry of Both Activation of and Interference With Multiple Transcriptional Pathways

Follow-up analyses of downregulated DEGs using the TF Perturbations Followed by Expression database identified multiple examples of the molecular mimicry of pathway activation and pathway interference gene expression signatures (GES) identified in fb-PMT-treated human AML cells (**Table 1**; **Supplementary Figure S7**). Notable examples of the fb-PMT-induced GES of transcriptional pathway’s activation include *RB1*, *IRF9*, *MAML1*, *RAP1A*, and *GATA4* pathways, and examples of the fb-PMT-induced GES of pathway’s interference include *MYC*, *HIF1A*, *TWIST1*, and *TFAP2C* pathways. Integrations of DEGs comprising fb-PMT-induced GES listed in **Table 1** identified a total of 25 genes, the differential expression of which appears to define molecular signals of either activation of or interference with transcriptional pathways in fb-PMT-treated human AML cells (**Supplementary Figure S7**). GSEA of genes comprising the 25- and 12-gene expression signatures validated their significance in defining observations of the molecular mimicry of transcriptional pathways’ activation and interference induced by fb-PMT treatment in human AML cells (**Table 2**; **Supplementary Figures S7–S9**). GSEA of all significant DEGs confirmed and extended these findings (**Supplementary Table S1**).

Interestingly, GSEA identified the *SNAI* transcriptional pathway as the most significantly enriched pathway of the molecular interference observed in K562 cells treated with fb-PMT among either down- or upregulated DEGs (**Supplementary Figure S10** and **Supplementary Tables S1, S2**). Additional examples of the specific genes and pathways of potential functional significance revealed by the GSEA of 233 genes downregulated in KG1a cells after fb-PMT treatment are shown in **Table 3** and **Supplementary Figure S9** and **Supplementary Table S3**. Of note, GSEA of the LINCS L1000 Ligand Perturbations database of upregulated genes revealed evidence of molecular interference with functions of multiple growth factors in human cancer cell lines.

#### GSEA of fb-PMT Effects on Gene Expression Revealed Signatures of the Molecular Interference With a Regulatory Crosstalk of Estrogen Pathway and Multikinase Transcriptional Matrix of Cell Cycle Progression

GSEA of databases of Ligand Perturbations from GEO focused on upregulated genes, and ligand perturbations from GEO focused on downregulated genes revealed multiple examples of molecular interference with transcriptional signaling induced by many endogenous ligands, among which the enrichment of genes implicated in estrogen signaling was particularly apparent (**Supplementary Figure S11** and **Supplementary Table S3**). Similarly, GSEA of the ARCHS4 Kinases Co-expression database revealed evidence of the molecular interference with functions of multiple kinases. GSEA of the integrated 69-gene signature of the fb-PMT interference with estrogen signaling in human AML cells identified 16 genes that appear implicated in transcriptional regulatory crosstalk of estrogens with multiple kinases in human tissues. Integration of 16 estrogen-regulated genes with 35 genes encoding kinases engaged in regulatory crosstalk in human tissues generated the 50-gene signature of estrogen pathway/multi-kinase gene expression regulatory matrix, which appears engaged in the cell cycle progression pathway. Intriguingly, GSEA of the 50-gene interference signature with estrogen-kinase regulatory matrix using the DisGeNET database of human disorders revealed that these genes were implicated in a remarkably broad spectrum of human malignancies (**Supplementary Figure S11** and **Supplementary Table S3**).

## Discussion

The present study assessed the efficacy of a novel thyrointegrin αvβ3 antagonist (fb-PMT) against human AML cells. fb-PMT proved to be a highly effective anticancer agent *in vivo*. Experiments using different human AML models in mice documented eradication of the leukemic cells’ engraftment after 3–4 weeks of continuous treatment with fb-PMT. Experimental therapy ON + OFF studies showed the significant efficacy of fb-PMT in preventing relapse, thus confirming the curative effects *in vivo* of fb-PMT treatment in clinically relevant animal models of human AML. The role of integrin in leukemogenesis was indicated by Yi et al., who reported that binding of leukemia cells to the bone marrow extracellular matrix (ECM) through integrins might influence drug response and the survival of leukemic cells (11). Integrin αVβ3 has been reported to be more expressed in AML cells especially CD34‐positive cells, monocytic leukemias, patient with NPM, and FLT3‐ITD (14).

Consistent with the previous reports on mechanisms of anticancer actions of thyrointegrin αvβ3 antagonists (30–35), genome-wide microarray screens reported here demonstrated that fb-PMT appears to exert its potent anticancer actions on human AML cells through the molecular interference mechanism with multiple signaling pathways supporting growth and survival of leukemic cells. We detected significant molecular signals of transcriptional interference with gene expression induced in human cancer cells in response to multiple growth factors such as epidermal growth factor (EGF), insulin-like growth factor-1 (IGF-1), transforming growth factor alpha (TGFA), and many others.

Other significant examples of the fb-PMT-induced GES of pathway’s interference include *SNAI MYC HIF1A*, *TWIST1*, and *TFAP2C.* Notably, inference of potential contribution to the fb-PMT anticancer activity of the interference with these pathways seems highly congruent with their known biological functions such as cell cycle control (*MYC*), survival and maintenance of stem cells (*HIF1A*, *TFAP2C*), and essential features of the malignant phenotype (*TWIST1*, *SNAI*) (36–49).

*SNAI* expression was a common target for both fb-PMT-treated cells (K562 and KG1 cells). The overexpression of *SNAI1* in leukemic cells is the key modulator of epithelial-to-mesenchymal transition (EMT), which have recently emerged as new players in the leukemogenesis. *SNAI* expression contributes to impaired differentiation and proliferation of leukemic cells. The effect of Snai1 has been reported to be mediated by interaction with the histone demethylase (LSD1; lysine-specific histone demethylase1), which modifies different gene expression (49, 50).

Consistently, examples of the fb-PMT-induced GES of transcriptional pathway’s activation include *RB1*, *IRF9*, *MAML1*, *RAP1A*, and *GATA4* pathways, known biological functions of which appear highly consistent with the hypothesis that activation of these pathways may contribute to fb-PMT anticancer activity (51–54).

Finally, consistent with our previous reports on the crosstalk between integrin αvβ3 and estrogen receptor α (ERα), which contributes to the induced proliferation of cancer cells (55–58), we found that fb-PMT interfered with estrogen signaling in human AML cells. The αvβ3 agonist (thyroid hormone) was associated with increased phosphorylation and nuclear enrichment of Erα (55). Hsiesh’s group reported that both T4 and estradiol (E2) caused nuclear translocation of integrin αv and phosphorylation of Erα in ovarian cancer cell line (SKOV-3). They successfully decreased the cell proliferation (by more than 60%) using specific ERα antagonist (ICI 182,780; fulvestrant), which blocks T4-induced ERK1/2 activation, ERα phosphorylation, proliferating cell nuclear antigen (PCNA) expression, and proliferation (55).

fb-PMT is an effective anticancer agent against solid tumors and hematological malignancies, with broad spectrum, potent anti-angiogenic activity against all known growth factors and other pro-angiogenesis stimuli (9, 59, 60–62). Collectively, preclinical findings of fb-PMT warrant its clinical investigation for the effective and safe management of AML.

## Conclusion

Our novel thyrointegrin αvβ3 antagonist, fb-PMT, represent a potential clinical candidate supported by its efficacy against human xenograft models of AML. Our genomic data demonstrated the potent anticancer actions on human AML through the molecular interference mechanism with multiple signaling pathways supporting growth and survival of leukemic cells. fb-PMT could have a broader application because it could be utilized, either alone or in combination with chemotherapeutic agents, to treat AML or other cancers.

## Data Availability Statement

The datasets presented in this study can be found in online repositories. The names of the repository/repositories and accession number(s) can be found below: https://www.ncbi.nlm.nih.gov/genbank/, GSE183772.

## Ethics Statement

Primary human AML cells (De novo AML 6373, harboring FLT3-ITD mutation) were collected by leukapheresis from AML patients at the University Hospital, University of Pennsylvania, with informed consents obtained per IRB (IRB protocol 703185). Mice were used in accordance with Public Health Service Policy on Humane Care and Use of Laboratory Animals and approved by the Albany VA Medical Center (Albany, NY, USA) IACUC (protocol number 545017).

## Author Contributions

SM designed the experiment and is the principal investigator. ND and ST conducted the experiment. ND did the data analysis. GG performed genomics and bioinformatics analyses, contributed to data interpretation and manuscript writing. ND contributed to the manuscript writeup and data interpretation. All authors contributed to the article and approved the submitted version.

## Conflict of Interest

SM holds several patents on anticancer compounds assigned to NanoPharmaceuticals LLC and founder of the company. GG is a consultant to NanoPharmaceuticals LLC.

The remaining authors declare that the research was conducted in the absence of any commercial or financial relationships that could be construed as a potential conflict of interest.

## Publisher’s Note

All claims expressed in this article are solely those of the authors and do not necessarily represent those of their affiliated organizations, or those of the publisher, the editors and the reviewers. Any product that may be evaluated in this article, or claim that may be made by its manufacturer, is not guaranteed or endorsed by the publisher.

## Supplementary Material

Supplementary Figure 1 Monitoring of blast cells in peripheral blood in engrafted transgenic mice. **(A)** K562-Luc (AML cell line) in control versus fb-PMT treated mice at different doses over 21 days during treatment and over 14 days post-treatment. fb-PMT at 1, 3, and 10 mg/kg, subcutaneous daily for 21 days, prevented blast cell expression/reproduction by 35%, 65%, and >95%, respectively, compared to controls. OFF treatment, fb-PMT (10 mg/kg) shows maintained remission. **(B)** Primary AML cells (6373-FlT3-ITD) cells in peripheral blood in control versus fb-PMT treated mice through 28 days and 14 days post-treatment. fb-PMT at 1, 3, and 10 mg/kg, subcutaneous daily for 28 days, prevented abnormal blast cell expression/reproduction by 54%, 75%, and 98.5%, %, respectively, compared to controls. fb-PMT treated mice (10 mg/kg) were in full remission after discontinuation of treatment for 2 weeks, (***P < 0.0001, **P < 0.001, *P < 0.01).

Supplementary Figure 2 Blood smear from transgenic mice engrafted with leukemic cells. **(A)** K562 cells in peripheral blood (PB) cells, control blood smears versus fb-PMT treated mice at different doses. Blast cells appeared in the blood smears of NSG-S mice after 10 days. After 21 days of fb-PMT daily subcutaneous injection, the control group showed many blast cells in the peripheral blood, while fb-PMT (1, 3, and 10 mg/kg) treated mice showed a significant decrease in the blast cells by 35%, 65%, and >95%, respectively. The decrease of blast cell percentage in the peripheral blood was dose-dependent. Left side shows blast cells in peripheral blood in fb-PMT treated animals at different doses ON 21 Days and ON+OFF treatment for 14 days and right side shows representative K562-Luc AML control blood smears versus fb-PMT (10 mg/kg) treated animal cells. Control group showed immature cells with prominent nucleoli (blast cells) in the peripheral blood. fb-PMT (10 mg/kg) treated animal showed segmented neutrophils with absent blast cells, after 21 days of daily subcutaneous injection. fb-PMT (10 mg/kg) maintained remission after 2 weeks discontinuation. **(B)** Primary AML cells (6373-FlT3-ITD), fb-PMT treated at 1, 3, and 10 mg/kg subcutaneous daily for 28 days and 14 days OFF treatment prevented blast cell expression/reproduction by 54%, 75%, and 98.5%, respectively, compared to controls. Right side shows representative image from control group with immature cells and prominent nucleoli (blast cells) in the peripheral blood where fb-PMT (10 mg/kg) treated animal shows segmented neutrophils with absent blast cells. OFF study, 14 days post-treatment, fb-PMT (10 mg/kg) showed successful maintained remission.

Supplementary Figure 3 IVIS scans of mice engrafted with AML K562-Luc and sacrificed after 23 days of fb-PMT (ON treatment). fb-PMT at 10 mg fb-PMT treatment associated with markedly reduced luminescent intensity.

Supplementary Figure 4 IVIS scans of different organs in transgenic mice engrafted with AML K562-Luc and sacrificed after 23 days of fb-PMT (ON treatment). fb-PMT at 1, 3 and 10 mg fb-PMT treatment, markedly reduced brain, lung, liver, and spleen infiltration with leukemic cells in dose dependent manner (***P < 0.0001).

Supplementary Figure 5 Evaluation of leukemic cells infiltration of spleen. **(A)** Evaluation of splenic infiltration of leukemic cells in transgenic mice engrafted with primary AML cells (6373-FlT3-ITD) control versus fb-PMT treated animal through 28 days and 14 days post-treatment. The splenic weight shows marked decrease in weight (80%) even with the low dose fb-PMT (1 mg/kg). ON+OFF groups showed maintained normal splenic weight in comparison to control, which may reflect successful prevention of engraftment. **(B)** Histopathological evaluation of splenic infiltration with primary AML cells in fb-PMT treated (10 mg/kg) versus control group. Upper row (control group) shows 90-95 % Infiltration with leukemic cells. Lower row (fb-PMT, 10 mg/kg) shows multiple normal lymphoid follicles with leukemic cells infiltration 5-10%.

Supplementary Figure 6 Monitoring of the weights of engrafted transgenic mice. **(A)** K562-Luc (AML cell line) mice group` body weights, control versus fb-PMT treated mice at different doses over 21 days during treatment and over 14 days post-treatment. **(B)** Primary AML cells (6373-FlT3-ITD) mice group` body weights, control versus fb-PMT treated mice through 28 days and 14 days post-treatment.

Supplementary Figure 7 Identification and characterization of the 25-gene fb-PMT-induced expression signature in K562 cells. **(A)** Integration into different functional categories of fb-PMT treatment-induced gene expression signatures (GES) as reported in **Supplementary Table S1**. **(B)** GSEA of 25-gene GES using the TF perturbations followed by expression database. **(C)** GSEA of 25-gene GES using the Gene perturbations from gene expression omnibus (GEO) database focused on up-regulated genes. **(D)** GSEA of 25-gene GES using the GEO database focused on down-regulated genes. Numbers shown next to the bar graphs report the values of Cumulative scores (Methods).

Supplementary Figure 8 Identification and characterization of the 25-gene fb-PMT-induced expression signature in K562 cells. GSEA of 25-gene GES using the Endogenous Ligands Perturbations from GEO databases focused on up-regulated **(A)** and down-regulated **(B)** genes. Numbers shown next to the bar graphs report the values of Cumulative scores (Methods).

Supplementary Figure 9 Examples of functionally-significant genes down-regulated by fb-PMT treatment in KG1A human AML cells. Stars designate known AML driver genes. Corresponding p values and fold-expression changes are reported in the **Supplementary Table S3**.

Supplementary Figure 10 Identification and characterization of the 39-gene SNAI pathway interference expression signature induced by fb-PMT treatment of K562 cells. GSEA of fb-PMT treatment targeted DEGs using TF perturbations followed by expression database identified 39-gene expression signature of molecular interference with SNAI1 pathway (see text). This GES harbors both down-regulated (cumulative enrichment score 5,385.7) and up-regulated (cumulative enrichment score 1,206.5) genes identified in SNAI1 knockout cells that are significantly enriched among fb-PMT targeted DEGs. GSEA of Ligand perturbations from GEO up-regulation (left) and Ligand perturbations from GEO down-regulation (right) databases.

Supplementary Figure 11 Identification and characterization of 50-gene fb-PMT signature of molecular interference with estrogen pathway/multi-kinase gene expression regulatory matrix of cell cycle progression. **(A)** 69-gene estrogen pathway interference signature harbors 16-gene estrogen-kinase crosstalk signature. **(B)** GSEA of the 50-gene interference signature with estrogen-kinase regulatory matrix using the DisGeNET database of human disorders. **(C)** 35-gene estrogen-kinase crosstalk regulatory matrix signature **(D)** Enrichr pathway analyses of the 50-gene interference signature with estrogen-kinase regulatory matrix identify the cell cycle progression pathway as the principal target.

## Abbreviations

AGCC, Affymetrix GeneChip Command Console Software; AML, acute myeloid leukemia; Akt, protein kinase B; BCR-ABL, breakpoint cluster region gene/Abelson proto-oncogene; BSA, bovine serum albumin; *CEBPA*, CAAT enhancer-binding protein alpha; DEGs, differentially expressed genes; ECM, extracellular matrix; Erα, estrogen receptor α; fb-PMT, fluorobenzyl polyethylene glycol conjugated tetraiodothyroacetic acid; FBS, fetal bovine serum; RPMI, Roswell Park Memorial Institute Medium; *FLT3-ITD*, FMS-like tyrosine kinase-3-internal tandem duplication; GEO, Gene Expression Omnibus; cDNA, complementary DNA; PBS, phosphate-buffered saline; HRP, horseradish peroxidase; GES, gene expression signatures; GSEA, gene set enrichment analysis; *HIF1A*, hypoxia inducible factor 1 subunit alpha; HSA, human serum albumin; IACUC, Institutional Animal Care and Use Committee; *IDH1/2*, isocitrate dehydrogenases 1 and 2; *IRF9*, interferon regulatory factor 9; IVIS, *in vivo* imaging systems; *MAML1*, mastermind-like transcriptional coactivator 1; MGED, microarray gene expression data; *NPM*, nucleophosmin; *RAP1A*, Ras-related protein Rap-1A; *RB1*, retinoblastoma protein-1; RGD, arginine–glycine–aspartate; s.c., subcutaneously; *SNA1*, snail family transcriptional repressor 1; TF, transcription factor; *TFAP2C*, transcription factor AP-2 gamma; TMB, tetramethylbenzidine; *TP53*, tumor protein p53; *TWIST1*, twist family BHLH transcription factor 1.
