# Supplementary figures and images for: Targeting Thyrointegrin αvβ3 Using Fluorobenzyl Polyethylene Glycol Conjugated Tetraiodothyroacetic Acid (NP751) in Acute Myeloid Leukemia
Source: Front Oncol. 2022 Jan 27;11:793810. doi: 10.3389/fonc.2021.793810 (PMC8828484; doi:10.3389/fonc.2021.793810)

## Slide 1
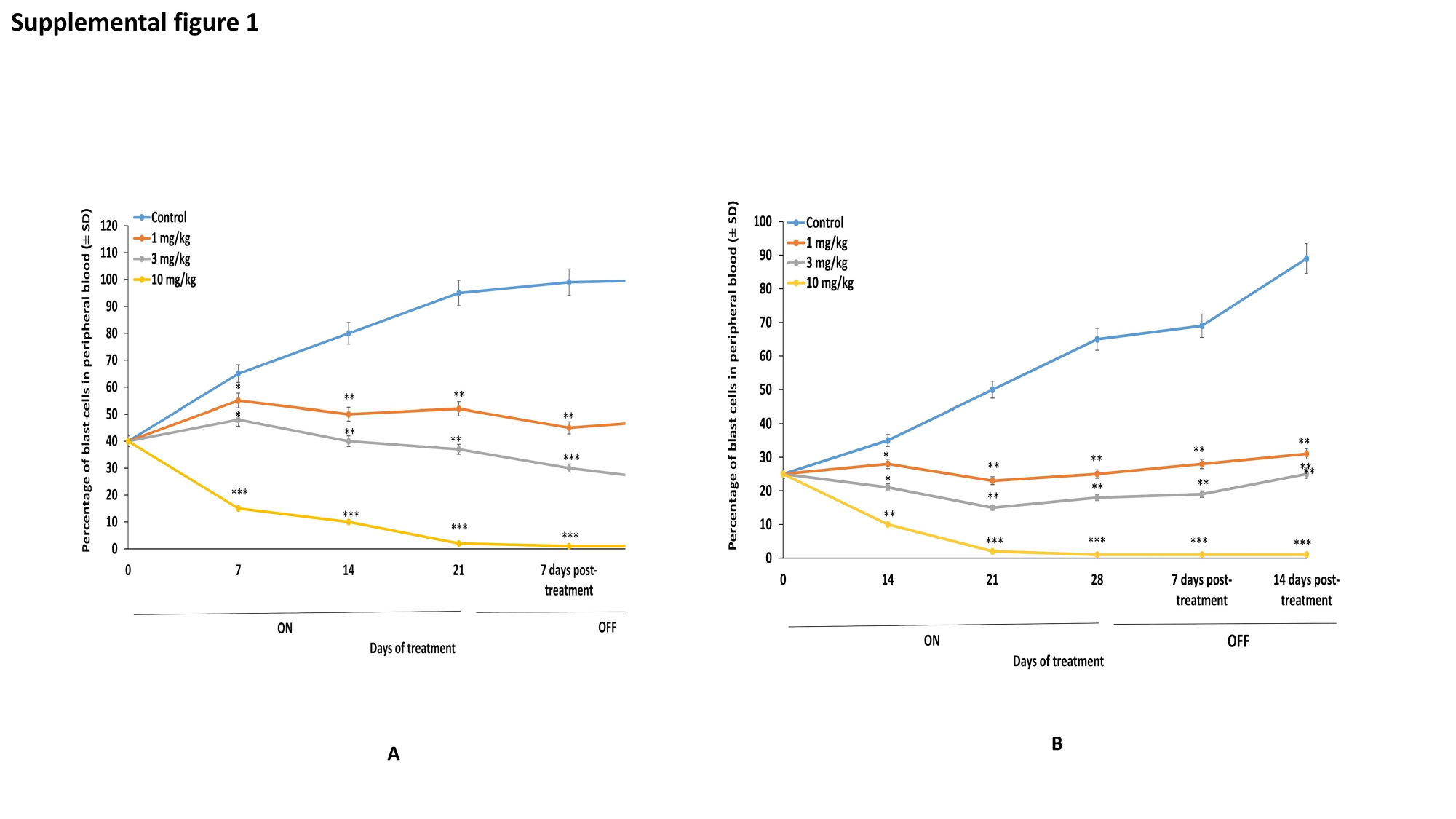

## Slide 2
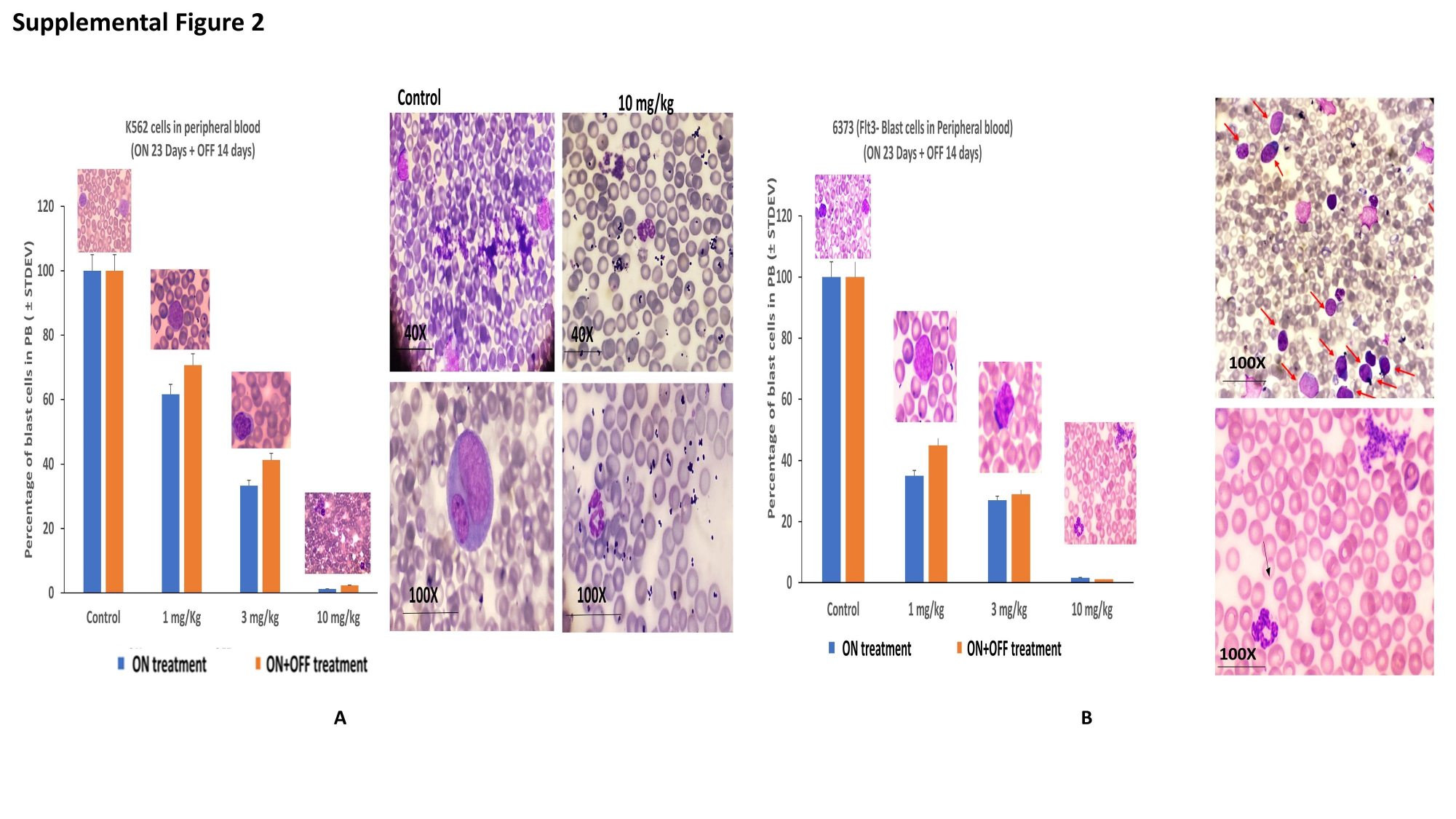

## Slide 3
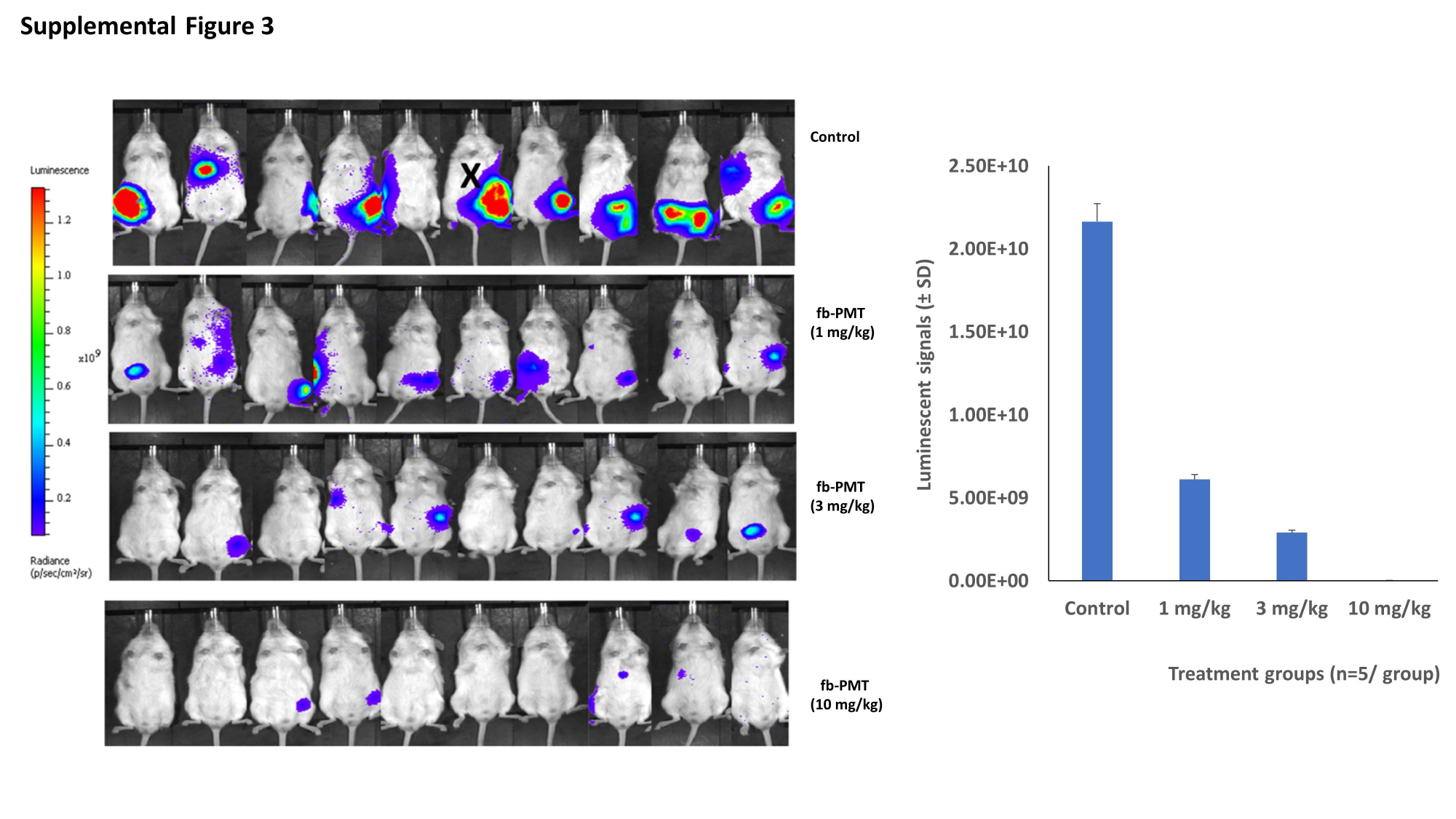

## Slide 4
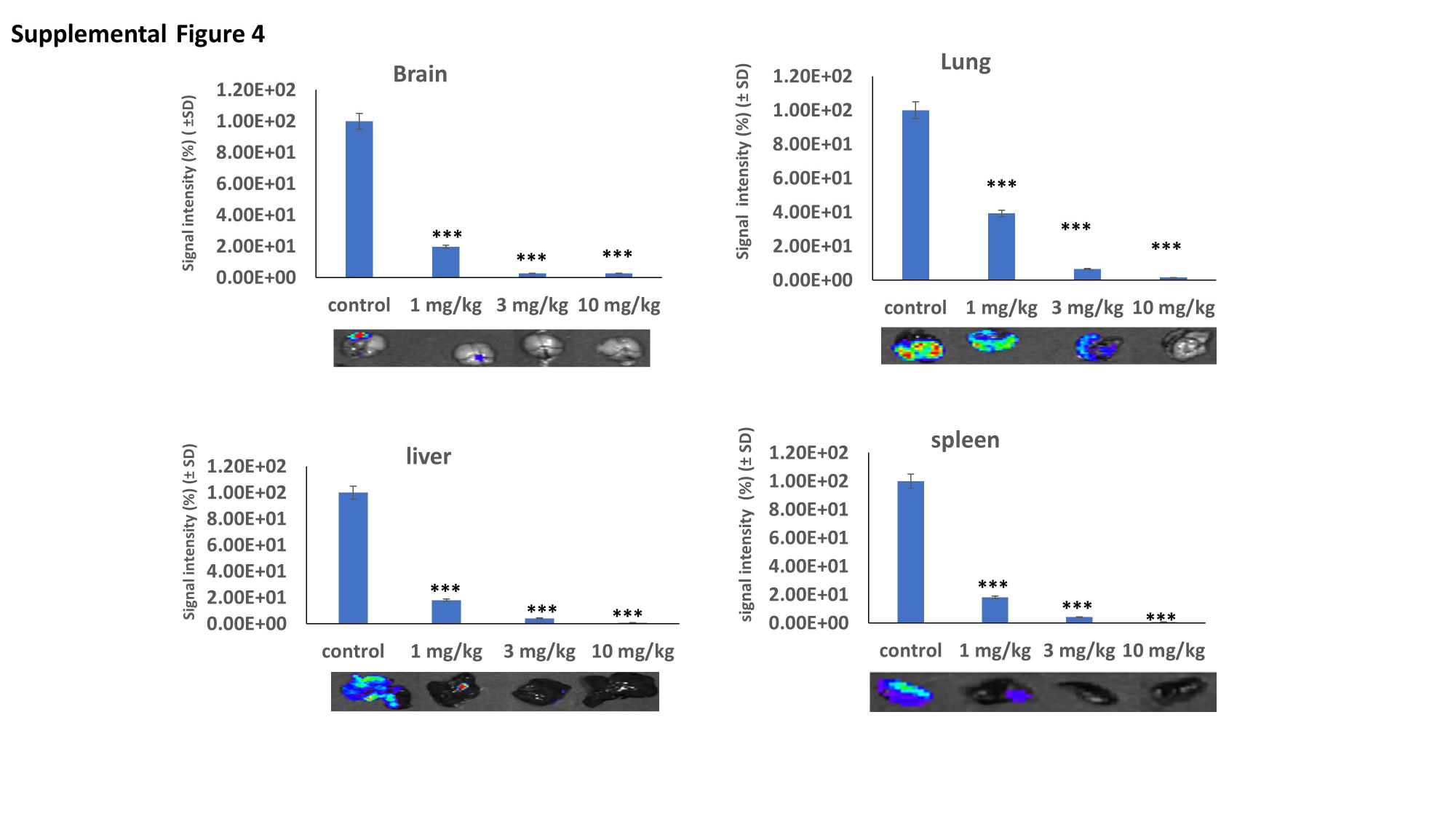

## Slide 5
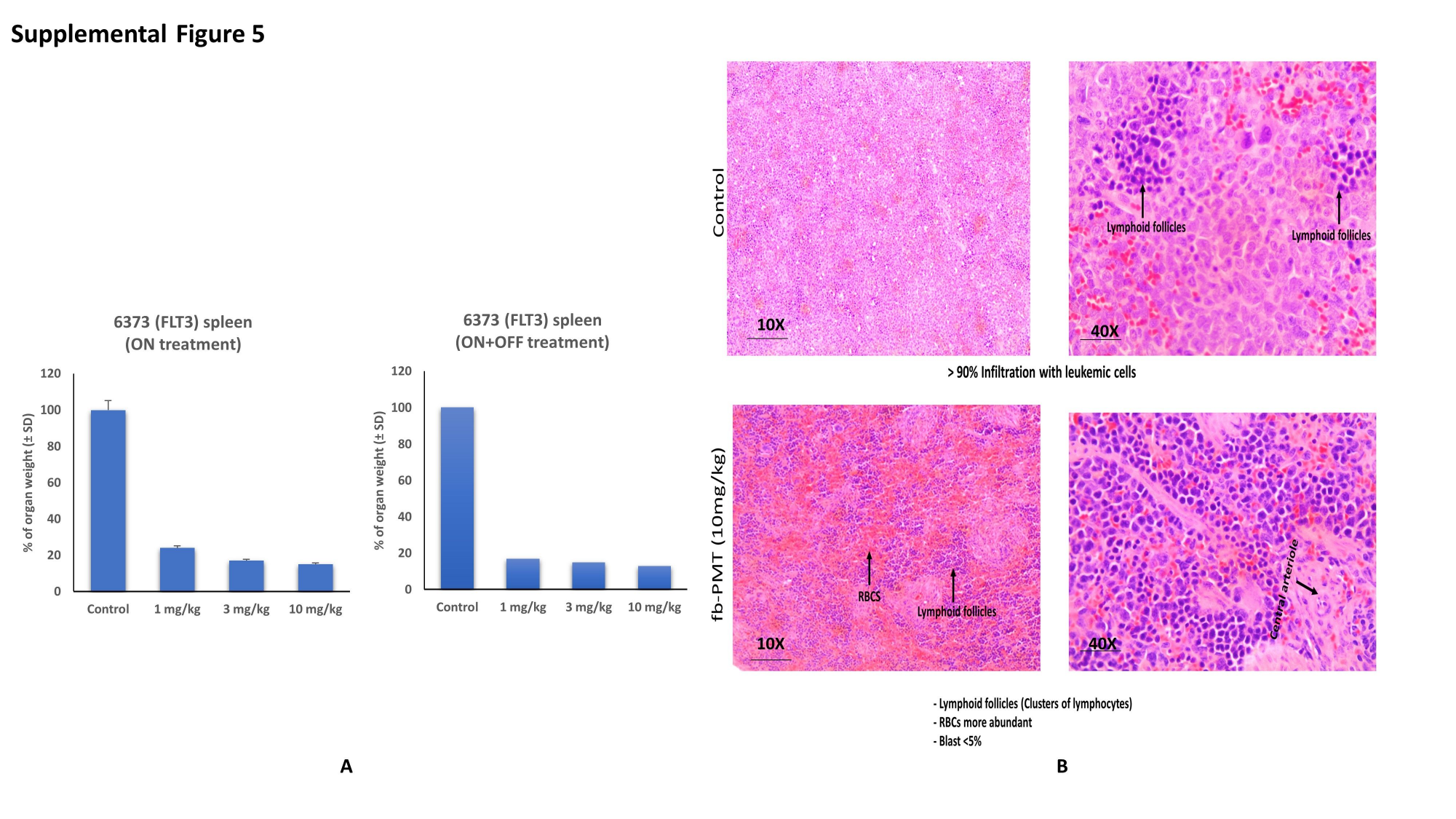

## Slide 6
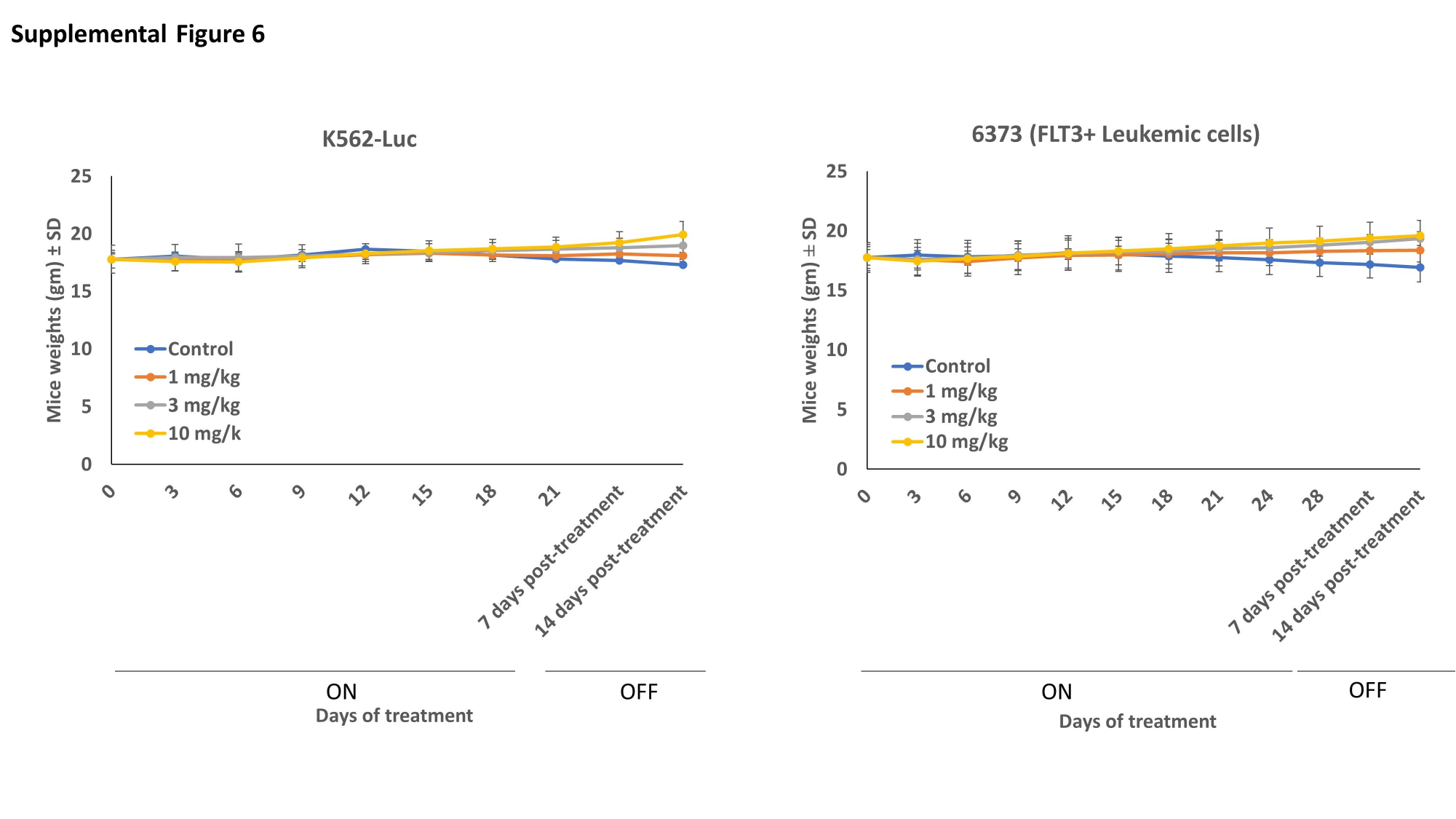

## Slide 7
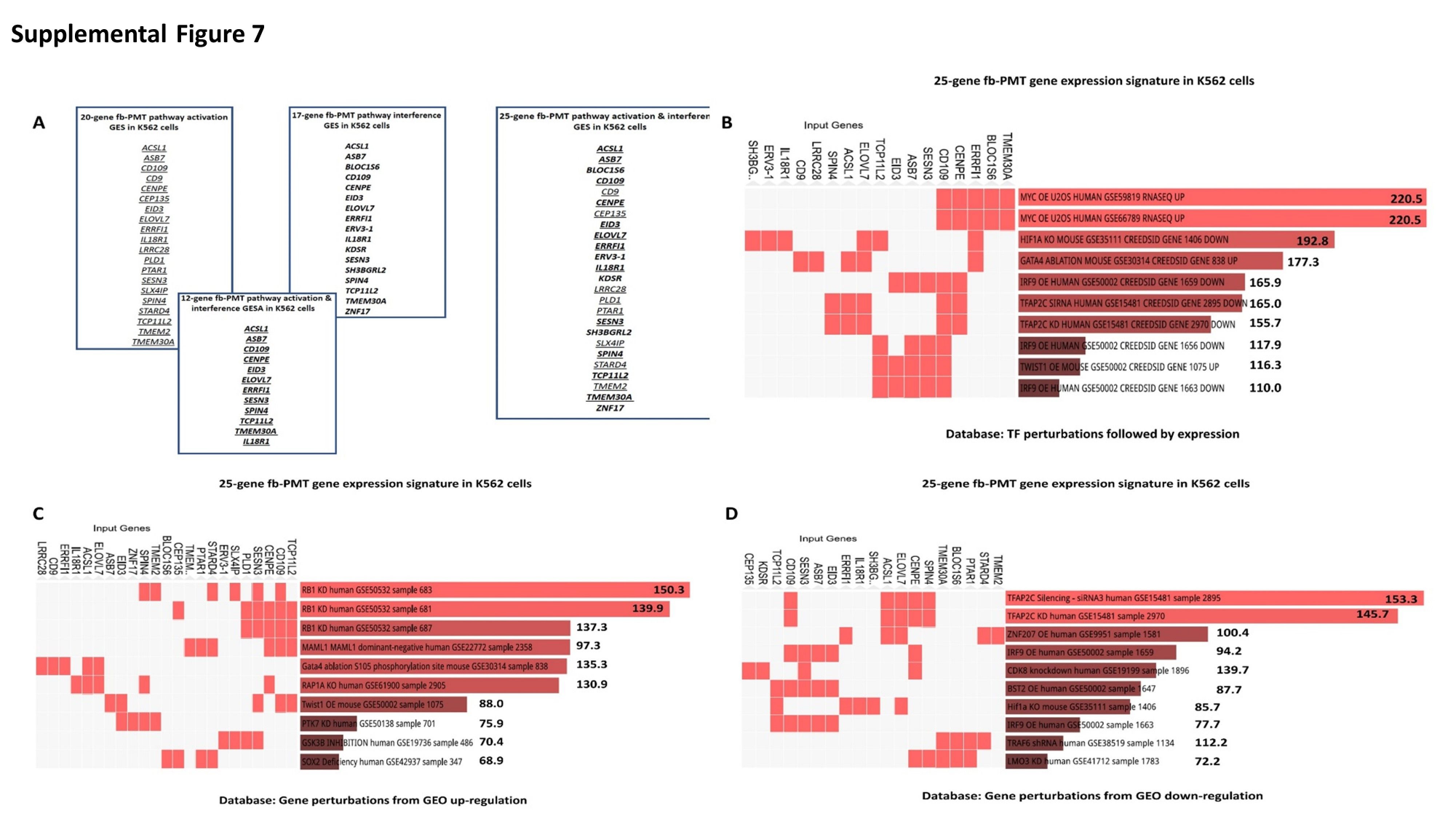

## Slide 8
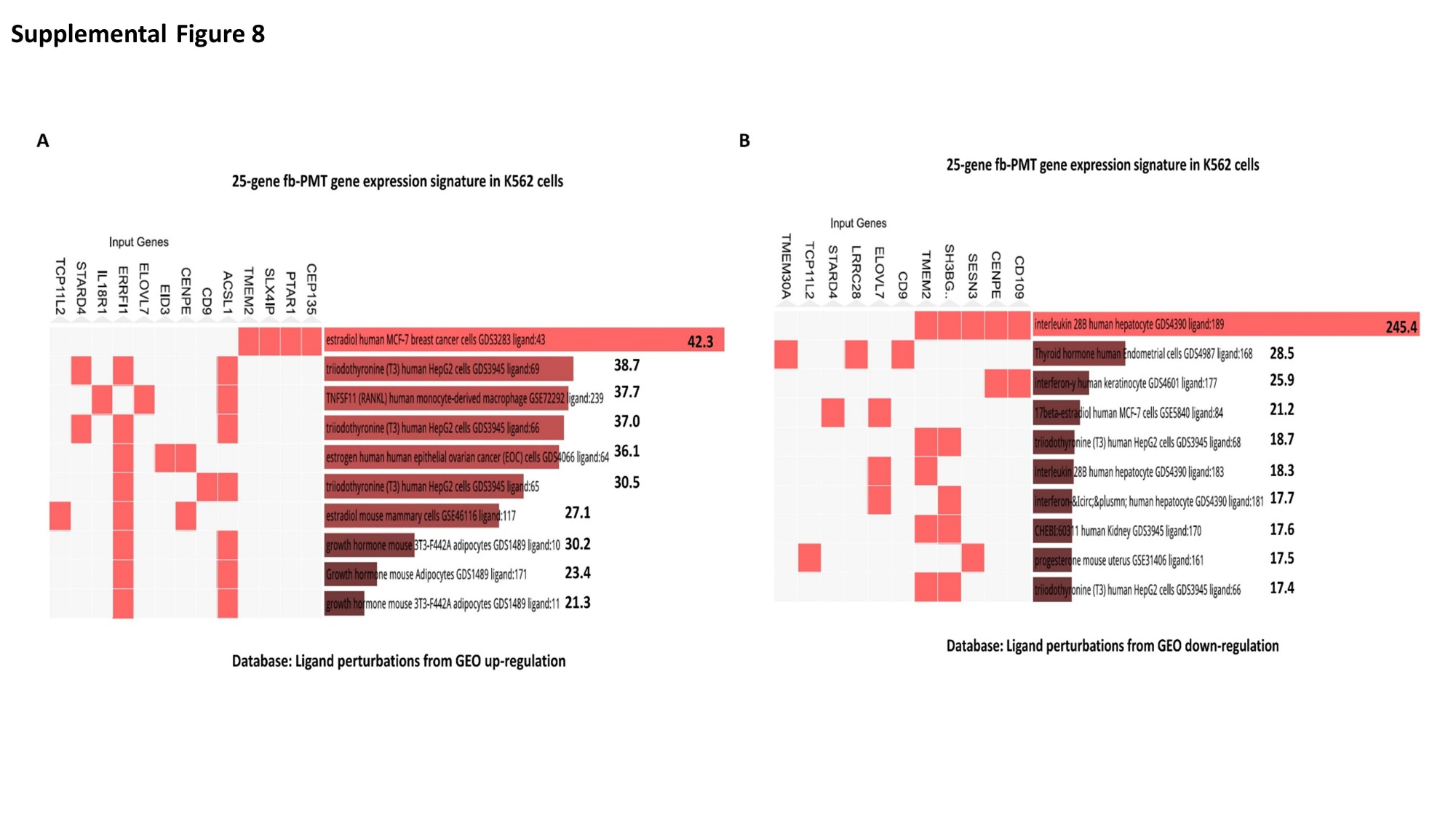

## Slide 9
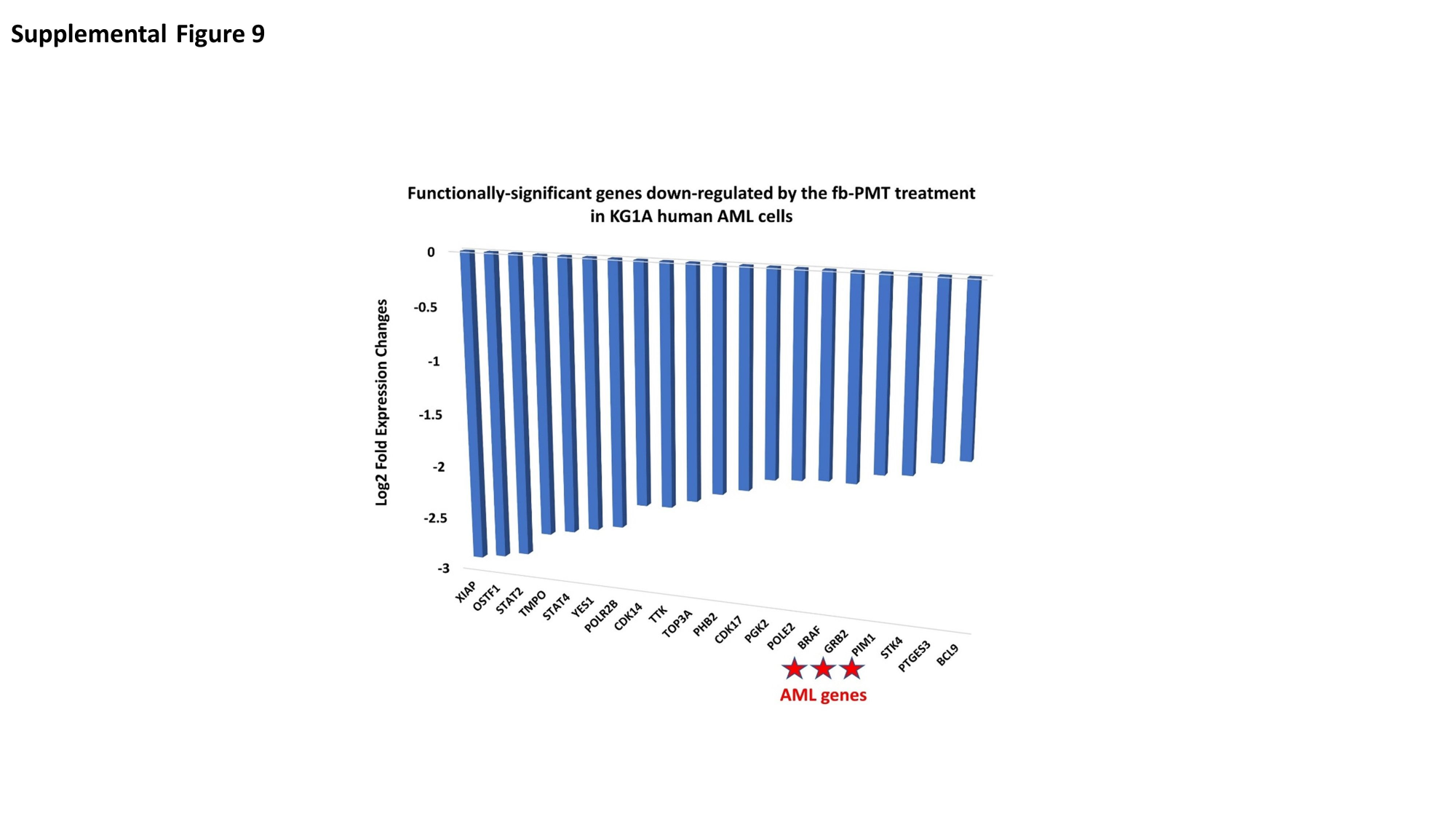

## Slide 10
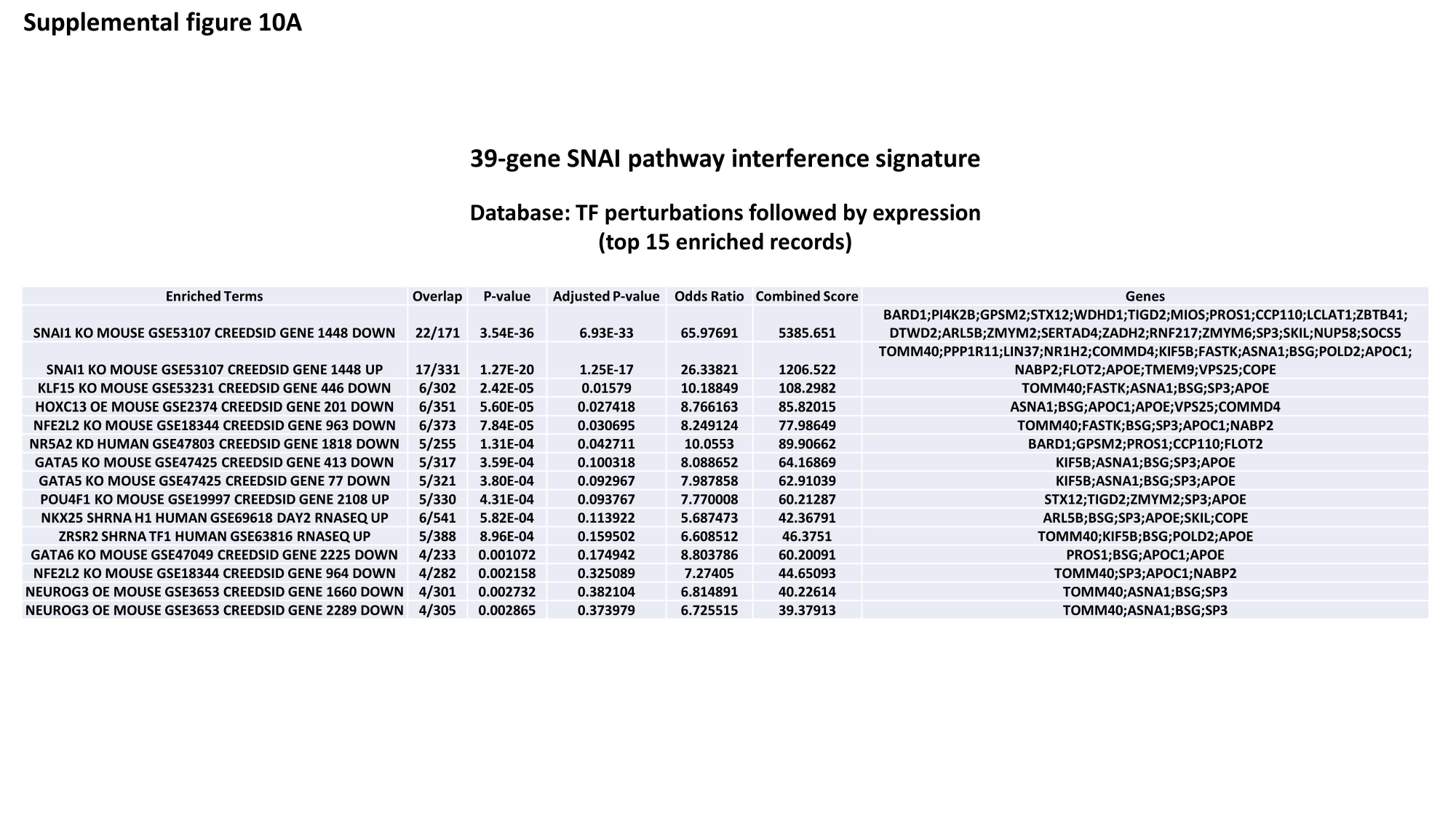

## Slide 11
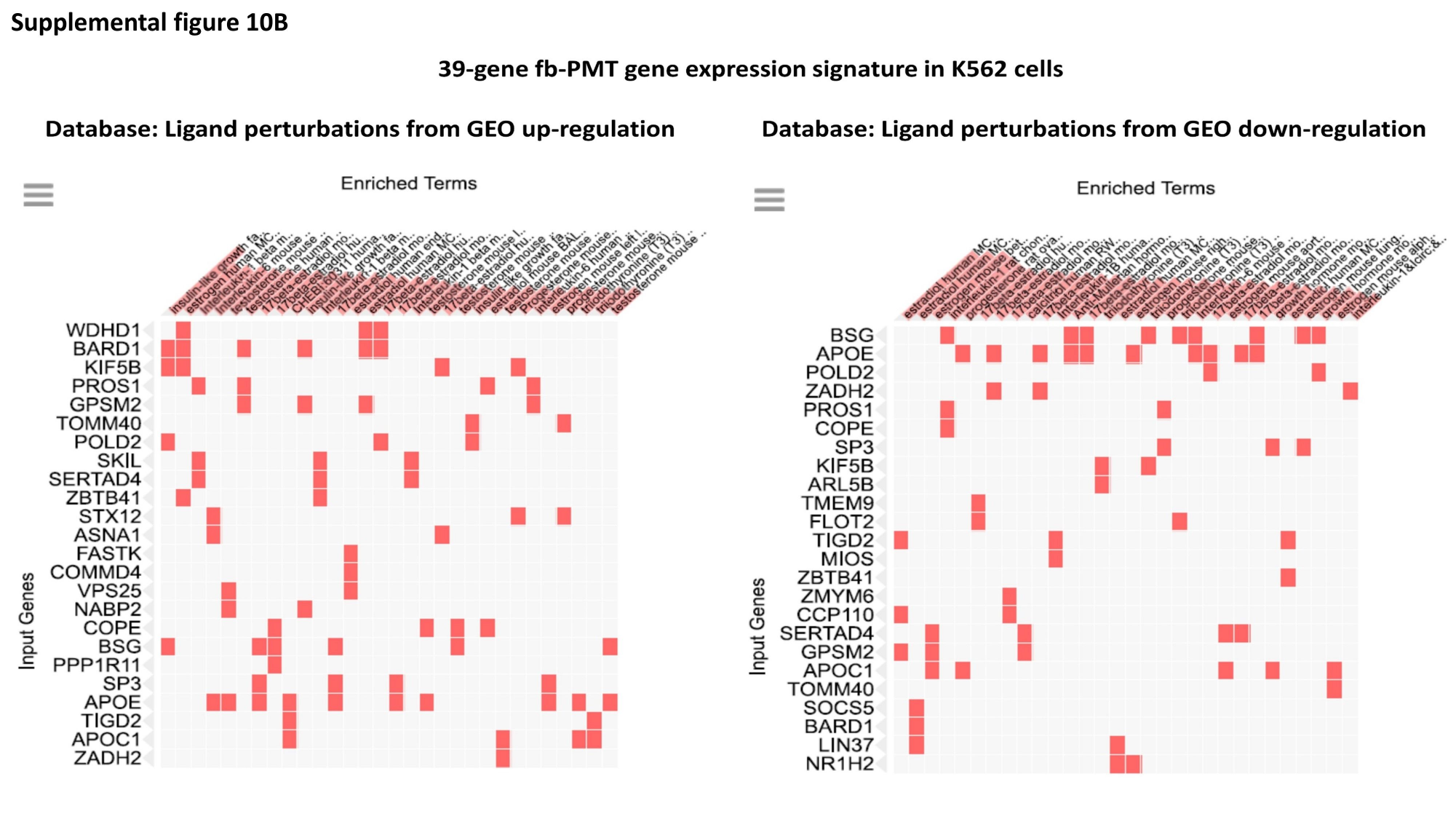

## Slide 12
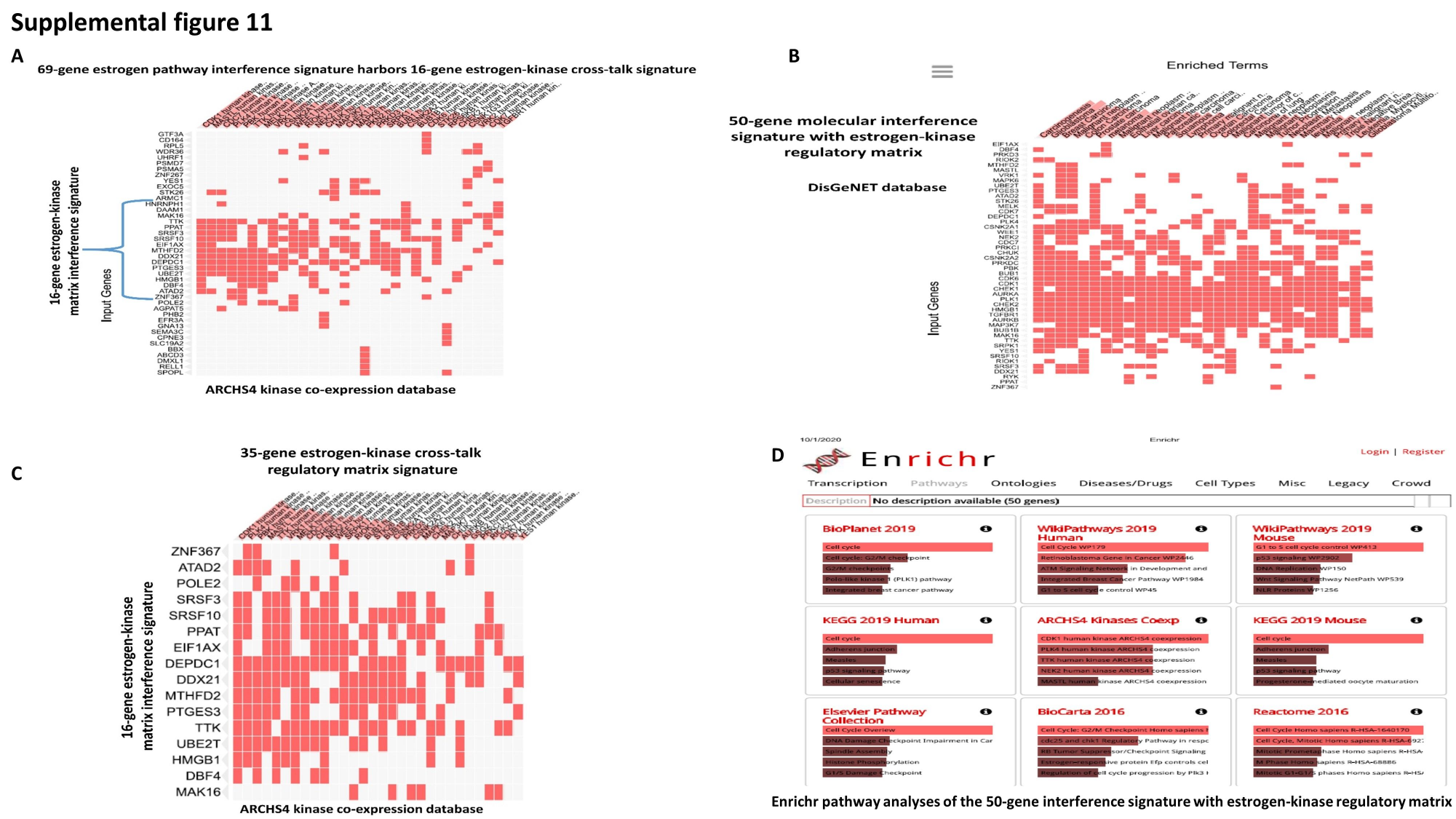

Supplement: Supplementary Figure 1 — Monitoring of blast cells in peripheral blood in engrafted transgenic mice. (A) K562-Luc (AML cell line) in control versus fb-PMT treated mice at different doses over 21 days during treatment and over 14 days post-treatment. fb-PMT at 1, 3, and 10 mg/kg, subcutaneous daily for 21 days, prevented blast cell expression/reproduction by 35%, 65%, and >95%, respectively, compared to controls. OFF treatment, fb-PMT (10 mg/kg) shows maintained remission. (B) Primary AML cells (6373-FlT3-ITD) cells in peripheral blood in control versus fb-PMT treated mice through 28 days and 14 days post-treatment. fb-PMT at 1, 3, and 10 mg/kg, subcutaneous daily for 28 days, prevented abnormal blast cell expression/reproduction by 54%, 75%, and 98.5%, %, respectively, compared to controls. fb-PMT treated mice (10 mg/kg) were in full remission after discontinuation of treatment for 2 weeks, (***P < 0.0001, **P < 0.001, *P < 0.01). [file DataSheet_1.zip › Supplementary Figures.pptx]
